# A Comparison of Different Sample Processing Protocols for MALDI Imaging Mass Spectrometry Analysis of Formalin-Fixed Multiple Myeloma Cells

**DOI:** 10.3390/cancers15030974

**Published:** 2023-02-03

**Authors:** Rita Casadonte, Jörg Kriegsmann, Mark Kriegsmann, Katharina Kriegsmann, Roberta Torcasio, Maria Eugenia Gallo Cantafio, Giuseppe Viglietto, Nicola Amodio

**Affiliations:** 1 Proteopath GmbH, 54296 Trier, Germany; 2 Department of Medicine, Faculty of Medicine and Dentistry, Danube Private University, 3500 Krems, Austria; 3 Institute of Pathology, University Hospital Heidelberg, 69120 Heidelberg, Germany; 4 Department of Hematology, Oncology and Rheumatology, Heidelberg University, 69120 Heidelberg, Germany; 5 Department of Experimental and Clinical Medicine, University Magna Graecia of Catanzaro, 88100 Catanzaro, Italy; 6 Laboratory of Cellular and Molecular Cardiovascular Pathophysiology, Department of Biology, Ecology and Earth Sciences (DiBEST), University of Calabria, Arcavacata di Rende, 87036 Cosenza, Italy

**Keywords:** cytospin, FFPE, imaging mass spectrometry, MALDI, sample preparation

## Abstract

**Simple Summary:**

Appropriate sample preparation is critical for the analysis of cell cultures with mass spectrometry. It is important not only to maintain clear and consistent morphological features, but also the local chemical composition of the sample. In this proof-of-concept study, we evaluated two different sample preparation procedures for proteomic analysis using imaging mass spectrometry (IMS) of formalin-fixed multiple myeloma (MM)-cultured cells. Cytospin preparation resulted in more peaks with a signal-to-noise ratio > 3 as compared to formalin fixation and paraffin embedding. Overall, IMS technology holds the potential to stratify different cell lines to address the identification of differentially expressed proteins. We propose this approach as an additional feasible method for proteomic investigation of MM cell lines, and potentially applicable to other tumor types.

**Abstract:**

Sample processing of formalin-fixed specimens constitutes a major challenge in molecular profiling efforts. Pre-analytical factors such as fixative temperature, dehydration, and embedding media affect downstream analysis, generating data dependent on technical processing rather than disease state. In this study, we investigated two different sample processing methods, including the use of the cytospin sample preparation and automated sample processing apparatuses for proteomic analysis of multiple myeloma (MM) cell lines using imaging mass spectrometry (IMS). In addition, two sample-embedding instruments using different reagents and processing times were considered. Three MM cell lines fixed in 4% paraformaldehyde were either directly centrifuged onto glass slides using cytospin preparation techniques or processed to create paraffin-embedded specimens with an automatic tissue processor, and further cut onto glass slides for IMS analysis. The number of peaks obtained from paraffin-embedded samples was comparable between the two different sample processing instruments. Interestingly, spectra profiles showed enhanced ion yield in cytospin compared to paraffin-embedded samples along with high reproducibility compared to the sample replicate.

## 1. Introduction

Mass spectrometry (MS)-based proteomics is an emerging approach to generate protein signatures from clinical samples for biomarker discovery [1,2,3,4]. In recent years, with the development of sample preparation methods and the advancement of MS instrumentation, many diseases have been studied by using cell analysis, such as pituitary [5], cervical [6], lung [7], ovarian [8], and breast cancers [9], as well as pleural effusion [10]. Desorption and ionization techniques, such as matrix-assisted laser desorption/ionization mass spectrometry (MALDI MS), have proven to be an excellent tool for molecular identification, providing detection of the analytes with no pre-selection and yielding simultaneous spatial information in combination with the imaging technology, that is imaging mass spectrometry (IMS) [11,12,13]. MALDI IMS is widely used to investigate tissue samples [14,15] and frequently applied in biology and clinical studies [16,17,18,19]; however, reducing the sample size from the tissue to the cell level frequently results in a decrease in chemical and morphological complexity, as well as in relative enrichment of unique analytes in the particular cell analyzed. Thus, cultured cells would produce a relatively higher signal throughout due to higher cell activity, which is not perturbed from the interaction of the tissue environment. The IMS technique not only preserves spatial information, but does not require sample homogenization, so less sample preparation is involved, and it also has the advantage of being non-destructive, allowing the sample to be used for further analytical tests [20,21]. 

In order to use IMS-based proteomics for the analysis of cell cultures in routine research and possibly in the clinical setting, the variability associated with the sample preparation steps that precede IMS analysis must be considered. 

We used multiple myeloma (MM) as a model system to establish an IMS experimental procedure for cultured cells.

Multiple myeloma (MM) is a B cell malignancy of clonal plasma cells (PCs) accumulating in the bone marrow (BM), characterized by an intricate genomic and epigenomic landscape. Despite the remarkable therapeutic achievements that have led to an increased extent and frequency of response with unprecedented outcome improvement, MM patients may eventually evolve towards a drug-resistant phase [22,23,24]. 

MS-based methodologies have proven to be a valuable diagnostic tool for minimal residual disease monitoring in plasma cell dyscrasias, including MM [25]. Additionally, proteomics approaches can be exploited in preclinical research to identify novel therapeutic targets in MM. In this regard, it must be underlined that, although cytogenetics and gene expression profiling (GEP) have advanced our understanding of MM, epigenetic and/or functional gene expression changes are not captured by such methodologies [26]. Therefore, proteomics remains the most valuable approach in the preclinical field to accurately portray the changes in protein expression underlying the malignancy. Accordingly, in the last decade we have observed an increasing number of studies analyzing the proteome of MM [27,28,29,30,31,32,33]. 

In order to further assess the clinical utility of MALDI MS, we optimized the sample preparation workflow for the proteomic investigation of MM cell cultures by comparing two different sample processing methods, including the use of the cytospin sample preparation, formalin fixation and paraffin embedding, and automated sample processing apparatuses. Herein, we present an optimized methodological approach, comparing two sample-embedding instruments using different reagents and with different processing times. We evaluated the peptide molecular extraction and the quality of mass spectral data obtained from fixed cell specimens with both procedures.

## 2. Materials and Methods

### 2.1. Cell Culture and Cell Fixation

**Figure 1:**
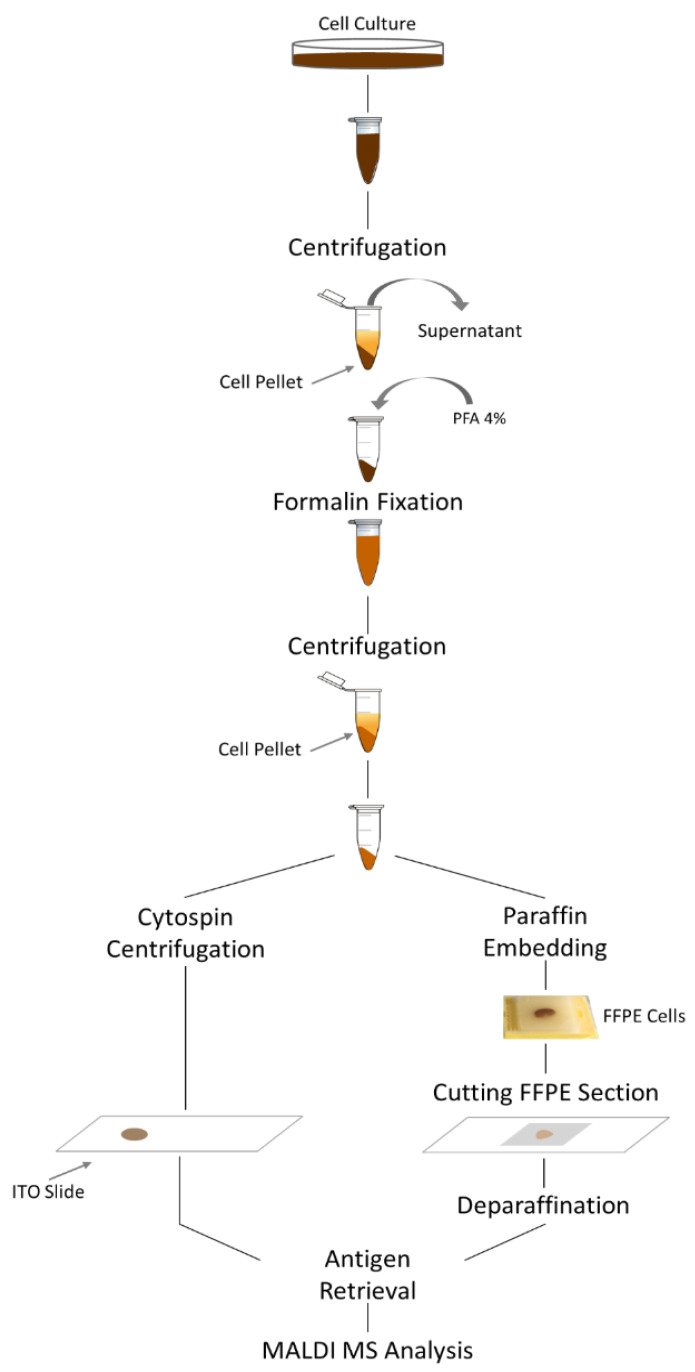
Representative workflow for formalin-fixed cell processing. Cell suspension was transferred to a centrifuge tube and concentrated by centrifugation for 10 min at 800 RCF. The cell pellet was resuspended with 4% paraformaldehyde and stored for approximately two weeks. Formalin-fixed cell suspension was centrifuged, and the paraformaldehyde was removed. One third of the cell pellet was resuspended in 70% ethanol, from which 150 µL was cytocentrifuged onto indium-tin oxide-coated (ITO) glass slides. The remaining cell pellet was processed to create a paraffin-embedded block. Then, 3 µm FFPE sections were mounted onto the ITO slide, dewaxed, and antigen retrieved together with the cytocentrifuged cells using heat-induced epitope retrieval combined with trypsin enzyme digestion. MALDI MS analysis was performed.

MM cell lines were cultured in RPMI-1640 medium (Gibco, Thermo Fisher, Monza, Italy) supplemented with 10% FBS and 1% P/S (Gibco) [23]. The AMO bortezomib-resistant cell line AMO BZB was obtained from parental AMO WT, as previously reported [34]. NCI-H929 GFP-luc were obtained from NCI-H929 cells after lentiviral transduction (Addgene#119816), according to standard procedures [35]. Each cell line was centrifuged at 3000 revolutions per min (RPM) for 5 min at room temperature and cell pellets were covered with 4% paraformaldehyde solution until further analysis (Figure 1).

### 2.2. Paraffin Embedding

For each cell line, the supernatant liquid was decanted in a separate tube and cell pellets were removed with a disposable Pasteur pipette and placed into a Tissue-Tek Paraform biopsy centralized cassette (Sakura Finetek Europe B.V., Umkirch, Germany). One third of the cell pellets was resuspended in 70% ethanol and stored at 4 °C until further use. The remaining cell pellets were then introduced in the automated tissue-processing machine (Sakura Tissue-TEK-Xpress-X120, Sakura Finetek Europe B.V.), where they were transferred from container to container to be processed. The pellet of the cell line “AMO BZB (8425)” was processed with a different automatic machine (Sakura Tissue-TEK-VIP, Sakura Finetek Europe B.V.) employing a similar solvent composition but a longer reagent incubation time. To create paraffin-embedded specimens, the tissue processors employ raised temperatures, a low-wattage microwave assistance (60 watts), and a vacuum infiltration technique to enhance processing and reduce the processing time from 8 h on a conventional processing platform to only 2.5 h when using Sakura Tissue-TEK-VIP, and to about 1 h for the Sakura Tissue-TEK-Xpress. Table S1 provides details of the various processing schedules used for dehydration, clearing, and the paraffin infiltration/incubation procedure. After hardening, paraffin blocks were stored in a dry, dark place at a temperature < 8 °C until use. 

### 2.3. FFPE Deparaffination and Antigen Retrieval

FFPE cell sections (3 µm) were mounted onto indium-tin oxide-coated (ITO) glass slides (Bruker Daltonik, Bremen, Germany) and dried at 37 °C overnight. Deparaffination occurred by heating the slides at 80 °C for 15 min, followed by incubation in xylene (2 × 5 min). Slides were dehydrated in 100% isopropanol, followed by graded ethanol (100%, 95%, 70%, 50%) and deionized water washes, for 3 min each. Antigen retrieval was performed by heating the slides at 95 °C for 20 min in 10 mM of Tris buffer pH 9 using a decloaking chamber (ZITOMED Systems GmbH, Berlin, Germany), followed by enzymatic retrieval using trypsin. Trypsin solution (0.025 µg/µL in 20 mM of ammonium bicarbonate) was applied on the samples with an automatic reagent sprayer (TM-Sprayer, HTX-Technologies, Chapel Hill, NC, USA) in 16 passes at 30 °C, with a 0.015 mL/min flow rate and a 750 mm/min velocity. Samples were then placed in a digestion chamber, prepared with saturated potassium sulphate solution, and incubated at 50 °C for 2 h.

### 2.4. Cytocentrifugation

The resuspended cell pellets were mixed by pipetting, and 150 µL of the cell suspensions was cytocentrifuged onto ITO-coated glass slides (Bruker Daltonik, Bremen, Germany) in a cytospin (Cellspin I Tharmac, Limburg an der Lahn, Germany) at 1200 rpm for 10 min. Slides were then incubated in 10 mM of Tris buffer pH 9, together with the dewaxed sections described above for the antigen retrieval process, and air-dried trypsin solution (0.025 µg/µL) deposition onto cytospin slides, and the digestion process occurred using the same method as described for the FFPE cell sections (Figure 1).

### 2.5. MALDI IMS Analysis

Digested samples were sprayed with a matrix solution (10 mg/mL of α-cyano-4-hydroxycinnamic acid in 70% acetonitrile, 1% trifluoroacetic acid) using the same sprayer apparatus in 4 passes at 75 °C, with a 0.120 mL/min flow rate and a 1200 mm/min velocity. MALDI MS data were acquired using a rapifleX MALDI Tissue-typer mass spectrometer (Bruker Daltonik) in positive-ion reflector mode in a mass range of 500–3200. Each mass spectrum was obtained by averaging signals from 200 laser pulses. External calibration was performed using the peptide calibration standard (angiotensin II, angiotensin I, substance P, bombesin, ACTH clip 1–17, ACTH clip 18–39, somatostatin 28) (Bruker Daltonik). All mass spectra were baseline-subtracted during acquisition with the TopHat algorithm. Following mass spectra acquisition, cells were stained with hematoxylin and eosin.

### 2.6. Data Analysis

Spectral data were pre-processed using FlexAnalysis 4.2, ScilS Lab software, version 2022b Pro (Bruker Daltonik), and R-statistical software, version 3.5.3 and 4.0.3, for total ion count (TIC) normalization, mass alignment, peak counting, and feature detection. Statistical analysis using Wilcoxon/Kruskal–Wallis tests and receiver operating characteristic curve (ROC) analysis was performed to measure the discrimination quality of the ion peptide features between the cell lines.

## 3. Results

Examination of the stained cells by light microscopy revealed a nice balance of nuclear and cytoplasmic stains in both types of cell sample processing methods, however cytospin-stained cells showed better cellular architecture and resolution of nucleoli (Figure S1).

**Figure 2:**
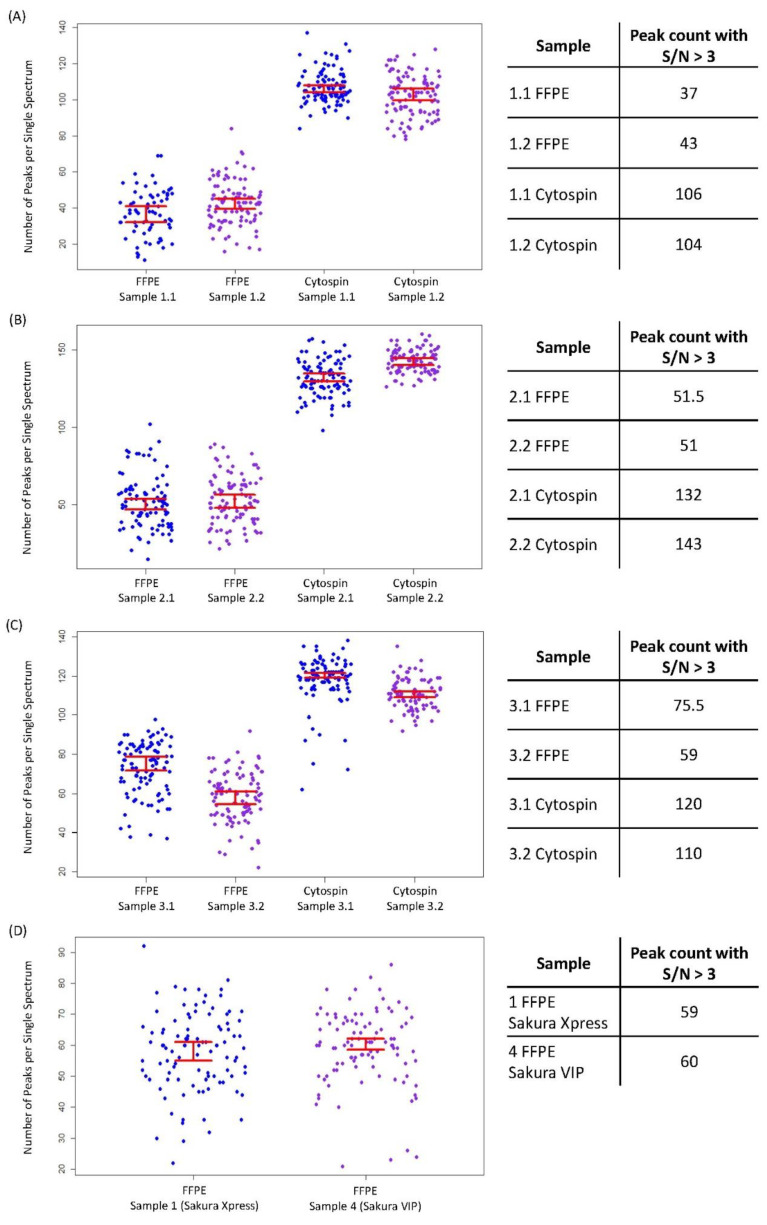
Comparative evaluation between FFPE and cytospin processed samples of AMO BZB ((**A**), sample 1), AMO WT ((**B**), sample 2), and H929 GFP/LUC ((**C**), sample 3) cell lines, and between replicate specimens (1.1 and 1.2, 2.1 and 2.2, 3.1 and 3.2). From each experiment, 100 random spectra were used to calculate the number of peaks per single spectrum with signal/noise ratio (S/N) > 3 and *m/z* > 500 < 3000. In each cell line, more peaks were detected from the cytospin compared to the FFPE cells. A similar number of peaks and low variance were achieved in each replicate. Peak count between cells processed with the two different tissue-processing apparatuses was similar (**D**). Each pixel in the graph corresponds to the number of peaks in a single spectrum. Bars in red represent one standard deviation.

High-quality MS signals were obtained from both FFPE and cytospin sample processing. A qualitative evaluation based on visual inspection and comparison of spectral data and molecular images showed better MS data quality with respect to cells prepared by cytospin with a higher ion yield (on average, by a factor of 2 in the *m/z* range 500–3000). The median total peak count and signal-to-noise ratios (S/N > 3) were used as quality parameters to compare all experiments. For each sample procedure, the overall amount of the peaks detected in the same cell line and for each individual replicate was similar (average number of peaks for FFPE = 40–67, for cytospin = 105–137). Nonetheless, the highest peak counts and lowest variance were achieved in all cytospin samples (Figure 2A–C). In order to confirm this result, the log2 of the intensity ratio of cytospin and FFPE for 269 common *m/z* peptide ions was computed using 69 spectra from each cytospin and FFPE dataset. No relevant imbalance of *m/z* between cytospin (*m/z* with log2 intensity cytospin/FFPE > 0 = 124) and FFPE (*m/z* with log2 intensity cytospin/FFPE < 0 = 145) was obtained, indicating that the higher number of peaks for cytospin is not related to a higher general peptide ion intensity (Table S2). However, it was observed that peaks in the low mass range (*m/z* < 800) were likely to have a higher intensity in cytospin than in FFPE, and conversely peaks in the high mass range (*m/z* > 800) had a higher intensity in FFPE than in cytospin (Figure S2A). This effect is due to measurement artifacts resulting in different noise characteristics in cytospin and FFPE samples. Cytospin samples presented a more sparsely packed cell layer than the FFPE sample (Figure S2B,C), causing an uneven coating of matrix crystals throughout the sample, particularly in those areas with few cells. This leads to different ionization efficiencies, where spectra are dominated by significant interferences of the background peak and contaminants arising from polymeric and matrix clusters (agglomerate of matrix molecules and alkali ions, typically sodium and potassium) in the low mass range (*m/z* < 800). In order to improve data quality and to prevent characteristic discrepancies between data collected from different samples, it Is recommended to extract spectra from regions of interest sufficiently far from areas with few cells. This would reduce the inclusion of the background interference signal in the dataset, thus further improving the quality of the analysis. 

The median total peak count from cells embedded with the automatic Sakura Xpress system (*n* = 59) was similar to the one obtained from cells embedded with the Sakura Vip apparatus (*n* = 60; Figure 2D). Peptide profiles obtained from the two FFPE cell blocks were similar (Figure S3), however the mass intensity for the comparable peaks was higher in the Sakura Xpress compared to the Sakura Vip procedure (Figure S3A).

**Figure 3:**
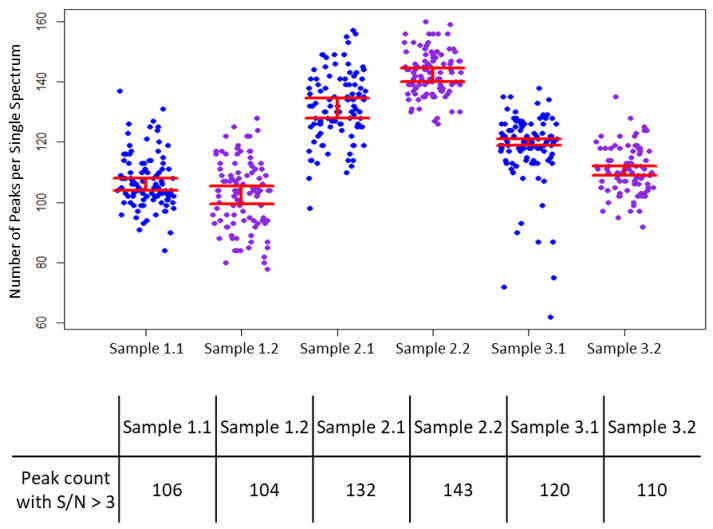
Peak count comparison from cytospin processed sample 1.1 with its duplicate 1.2 (AMO BZB), sample 2.1 and its duplicate 2.2 (AMO WT), and sample 3.1 with its duplicate 3.2 (H929 GFP/LUC). From each sample, 100 random spectra were used to calculate the number of peaks per single spectrum, with S/N > 3 and *m/z* > 500 < 3200. Sample 2 showed the highest number of peaks (peaks = 132 in replicate 2.1, peaks = 143 in replicate 2.2) compared to sample 1 (peaks = 106 in replicate 1.1, peaks = 104 in replicate 1.2) and sample 3 (peaks = 120 in replicate 3.1, peaks = 110 in replicate 3.2).

Considering the quality of the MS data recovered from cytospin procedure, the peak counts of the three cytospin cell lines were plotted for comparison using the same parameters (*m/z* range 500–3000, S/N > 3). The number of peaks per single spectrum was high in the AMO WT cells, with a median peak count of 132 in replicate 1 (Figure 3, sample 2.1) and 143 in replicate 2 (Figure 3, sample 2.2). Similar results were observed when MS spectral averages of cytospin cell lines were compared.

**Figure 4:**
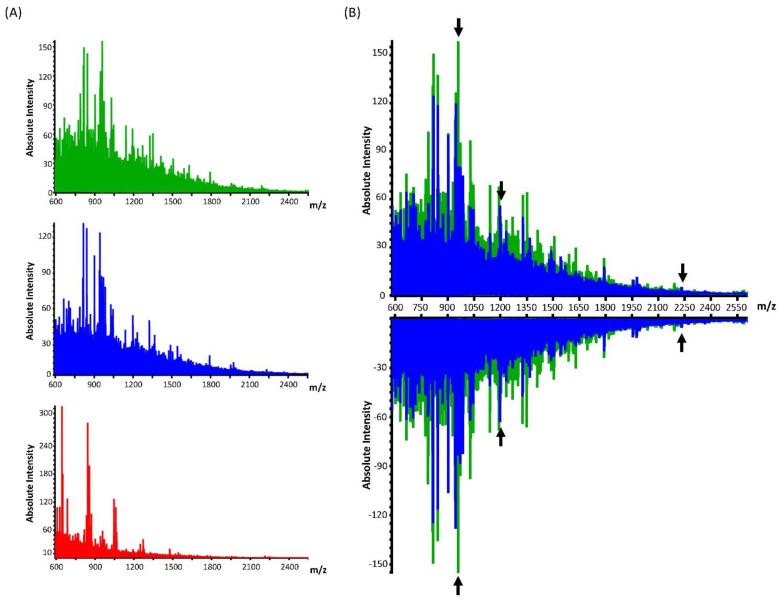

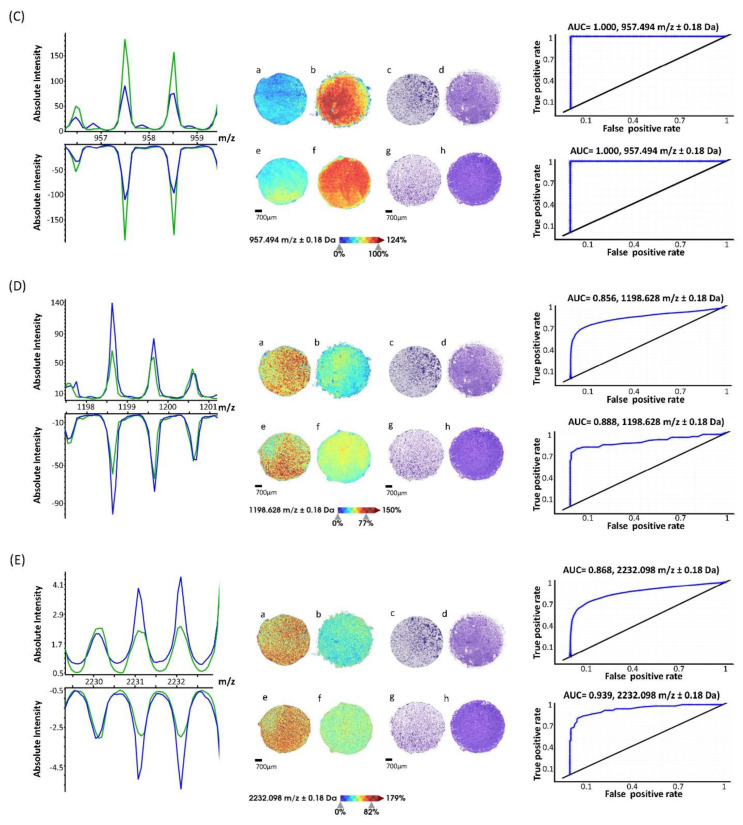
Comparison of peptide MS profiles of different cell lines processed with cytospin. (**A**) MALDI mass spectrometry spectra of the cell lines AMO WT (green), AMO BZB (blue), and H929 GFP/LUC (red). (**B**) MALDI MS overlay spectra of cell lines AMO BZB (blue) and AMO WT (green) in replicate 1 (spectra up) and replicate 2 (spectra down) samples. Representative peak discriminators, indicated with black arrows, are shown in enlarged areas for *m/z* 957.494 ((**C**), spectra left), showing high intensity in AMO WT cells (spectrum in green), 1198.628 ((**D**), spectra left), and 2232.098 ((**E**), spectra left), which show high intensity in the AMO BZB (spectrum in blue) cells. Ion density maps of these representative peptide peaks are shown in the AMO BZB cells for *m/z* 957.494 ((**C**)−a, replicate 1; (**C**)−e, replicate 2), *m/z* 1198.628 ((**D**)−a, replicate 1; (**D**)−e, replicate 2), and for *m/z* 2232.098 ((**E**)−a, replicate 1; (**E**)−e, replicate 2). Likewise, the ion density map of the same peptides is shown in the AMO WT cells for *m/z* 957.494 ((**C**)−b, replicate 1; (**C**)−f, replicate 2), *m/z* 1198.628 ((**D**)−b, replicate 1; (**D**)−f, replicate 2), and for *m/z* 2232.098 ((**E**)−b, replicate 1; (**E**)−f, replicate 2). Hematoxylin and eosin staining of the cytospin AMO BZB ((**C**)−c, (**D**)−c, (**E**)−c replicate 1; (**C**)−g, (**D**)−g, (**E**)−g replicate 2), and AMO WT ((**C**)−d, (**D**)−d, (**E**)−d replicate 1, (**C**)−h, (**D**)−h, (**E**)−h replicate 2) cells after MALDI MS analysis is represented. The color bar provides a visual map of the intensities (in arbitrary units) of the ion peptide images to a corresponding rainbow gradient colormap. Scale bars indicate 700 µm. For each peptide, the area under the ROC curve (AUC) is shown, with values > 0.7 ((**C**–**E**), right diagrams) indicating a good discrimination quality of those peptides between the two different cell lines.

It was also tested whether MALDI IMS could generate spectra from cytospin cells of sufficient quality to distinguish between the three cell lines (AMO WT, AMO BZB, H929 GFP/LUC). Peptide profiles of AMO WT and AMO BZB shared many peaks compared to the cytospin H929 GFP/LUC, whose MS data showed a different molecular profile trend (Figure 4A). An overall lower peptide signal intensity in AMO BZB (Figure 4B, spectrum in blue) compared to the AMO WT (Figure 4B, spectrum in green) cell line across the whole mass range was observed in both replicate samples (Figure 4B, spectra up for replicate 1; spectra down for replicate 2). Several peptide ions were found to be differentially expressed, exhibiting a weight statistical significance evaluated with the Wilcoxon/Kruskal–Wallis tests (PWKW < 0.001) and area under the ROC curve (AUC) > 0.7. 

A list of the most prominent and intense peptide peaks was compiled together with the average intensities, AUC, and PWKW *p*-values (Table 1). Specifically, 10 peptide peaks have been found as overexpressed and 16 peptides as under-expressed in AMO BZB compared to AMO WT (Table 1). A spectral and cell map view of some representative major peptide discriminators is shown in Figure 4C–E. These peptide profiles were found to be highly reproducible with little variation within the two types of cells.

## 4. Discussion

In this proof-of-concept study, two different sample procedures, including the use of the cytospin sample preparation and automated sample processing apparatuses, were evaluated for the analysis of MM cell cultures by imaging mass spectrometry. Hematoxylin and eosin revealed good-quality staining in both cell procedures based on visual inspection. However, a better cellular architecture as well as a better resolution of nucleoli in cytospin cells was observed, suggesting that the relatively rough conditions of high temperature, alcohol dehydration, and pressure that an automatic tissue processor uses for cell processing may lead to cell shrinkage and distortion [36]. 

The cytospin procedure of formalin-fixed MM cells resulted in the recovery of a higher number of peptides when compared with the paraffin-embedding process. It must be noted that the paraffin-embedding procedure requires some steps where cells undergo a series of alcohol treatments with raised temperatures and vacuum/pressure cycling before infiltration with paraffin. While this environment helps in the penetration and fluid exchange, and reduces the processing time, it may cause protein loss or protein alteration. Indeed, organic solvents lead to a severe loss of cell content, attributed to the removal of lipids and thus the loss of membrane integrity [37]. In addition, elevated temperatures interfere with the secondary structure of the proteins, leading to the unfolding, denaturation, and rearrangement of protein molecules responsible for protein aggregation [38,39]. Thus, alterations in the oligomeric state of a protein may result in a decrease of the accessible catalytic sites for enzymes such as trypsin, which was used here for the epitope retrieval. This can make proteins inaccessible for proteomic analysis and led us to speculate that the cytospin preparation method, which involves only centrifugation, may allow for less loss of protein information or a better demasking. A disadvantage of the cytospin technique is that long-term storage of cells is possible only by freezing in liquid nitrogen and storing them in an ultra-cold freezer at lower than −80 °C. This involves continuous snap freezing and thawing procedures when further studies need to be carried out, as well as expensive cold-storage logistics. In the FFPE blocks, cells are stable and can be easily archived for decades at room temperature. However, the efficacy of FFPE cell blocks in providing material available for further analysis has major disadvantages that occur during paraffin embedding, which include, in addition to the loss of protein information, artifacts of cellular shrinkage and distortion, and loss of cytological details.

Due to the higher molecule signal as compared to the FFPE method, the cytospin method was chosen to compare mass spectral data of the cell cultures. As expected, AMO WT and AMO BZB cells, which originate from the same cell line, displayed similar mass spectral profiles compared to H929 GFP/LUC. Nonetheless, after a deep evaluation, the method enabled the discrimination between AMO WT and its bortezomib-resistant isogenic counterpart, AMO BZB, suggesting that MALDI MS analysis is in principle suitable to differentiate even cell lines with similar genetic backgrounds but different drug sensitivities. Specifically, we observed that AMO BZB cells showed an overall decreased peptide profile versus AMO WT cells. This is in agreement with recent evidence demonstrating a differential proteomic profile, with downregulation of specific proteins, in proteasome inhibitor-resistant compared to sensitive MM cell lines [40,41]. However, follow-up studies increasing the size of samples are mandatory to address this biological issue, which is beyond the aim of the present work.

Comparison of the average relative intensities of the *m/z* ion peptides between AMO WT and AMO BZB cells revealed a total of 26 distinct *m/z* features that had weight statistical significance (area under ROC ≥ 0.7, ≤0.3; Wilcoxon and Kruskal–Wallis *p*-values ≤ 0.001). Sixteen *m/z* features were overexpressed in AMO WT, from which two features (*m/z* = 951.43, 2189.889) showed more than a two-fold intensity difference between the two cell lines. Ten features were instead overexpressed in AMO BZB cells, where three features (*m/z* = 502.24, 521.3, 549.27) exhibited more than a four-fold intensity difference. The identification of the *m/z* species making these specific signatures is beyond the scope of this study because they need validation in a larger dataset. Nevertheless, these results could be reproduced on separate experiments and on a different day. Nearly identical spectra as well as equivalent peak expression were obtained in the replicate experiments, where segregation of the two different cell lines, AMO WT and AMO BZB, following AUC analysis was also obtained. Such a highly reproducible technique could be applied to other types of cells and has the potential to be a useful tool for the stratification of cell lines based on their peptidomic profile, with the goal of rapidly identify novel potential tumor molecular signatures. 

## 5. Conclusions

This proof-of-concept study indicates that fixation in formalin followed by centrifugation in cytospin resulted in the recovery of higher numbers of peptides compared with fixation combined with paraffin embedding. It was also shown that IMS technology holds the potential to stratify cell lines of MM, including those with a similar genetic background but different drug sensitivity. We propose this approach as an additional method for proteomic analysis of MM samples, potentially applicable to other tumor types. The continued development of proteomics and its integration with genomic studies is expected to provide valuable information for the detection of molecular mechanisms and target proteins involved in MM onset and progression.

## Figures and Tables

**Table 1:** Statistical analysis revealed differentially expressed *m/z* species between AMO BZB and AMO WT cell lines. Average intensities, AUC, and *p*-values were combined from replicate experiments.

*m/z*	Avg. Intensity AMO BZB	Avg. Intensity AMO WT	AUC	Wilcoxon Rank Sum *p*-Value	Kruskal–Wallis *p*-Value	Log-Fold-Change
502.2	1579.8	88.7	1.00	0	0	4.16
521.3	137.2	32.9	0.99	0	0	2.06
530.3	455.8	306.2	0.78	0	0	0.57
549.3	359.4	67.0	0.99	0	0	2.42
648.3	101.5	138.1	0.02	0	0	−0.44
700.4	116.5	139.2	0.02	0	0	−0.26
717.3	101.1	135.6	0.01	0	0	−0.42
773.4	91.2	162.1	0.00	0	0	−0.83
781.4	83.5	139.7	0.01	0	0	−0.74
815.4	104.1	171.2	0.00	0	0	−0.72
904.4	63.3	116.1	0.00	0	0	−0.87
913.5	74.3	113.1	0.00	0	0	−0.61
927.5	72.2	141.0	0.00	0	0	−0.97
951.4	68.2	154.5	0.00	0	0	−1.18
957.5	188.9	318.7	0.00	0	0	−0.75
991.5	61.3	111.3	0.00	0	0	−0.86
1018.5	71.6	120.9	0.00	0	0	−0.76
1154.5	59.5	104.7	0.00	0	0	−0.82
1198.6	157.6	110.5	0.88	0	0	0.51
1955.0	35.5	27.3	0.74	0	0	0.38
2189.9	8.3	18.1	0.01	0	0	−1.13
2231.1	16.5	11.3	0.87	0	0	0.54
2232.1	17.6	11.9	0.90	0	0	0.56
2565.2	9.1	6.3	0.79	0	0	0.54
2585.3	8.2	10.0	0.23	2 × 10^−14^	2 × 10^−14^	−0.28
2769.4	7.0	5.3	0.72	8 × 10^−14^	8 × 10^−14^	0.39

## Data Availability

The data presented in this study are available upon reasonable request to the corresponding author.

## Supplementary Materials

The following supporting information can be downloaded at: https://www.mdpi.com/article/10.3390/cancers15030974/s1, Figure S1: HE-stained cells are represented for comparison between cells treated with the cytospin procedure and cells embedded in paraffin. Figure S2: Peak intensity distribution in cytospin and FFPE samples through the *m/z* range 500–2000. Figure S3: Peptide profiles and principal component analysis (PCA) obtained from two FFPE cell blocks prepared with two automatic processing systems. Table S1: Sample paraffin processing protocol. Table S2: Comparison of peptide ions’ (*m/z*) intensity between cytospin and FFPE datasets.

## References

[1] Fung E.T., Wright G.L., Dalmasso E.A. (2000). Proteomic strategies for biomarker identification: Progress and challenges. Curr. Opin. Mol. Ther..

[2] Zhou M., Conrads T.P., Veenstra T.D. (2005). Proteomics approaches to biomarker detection. Brief. Funct. Genom. Proteom..

[3] McDonald W.H., Yates J.R. (2002). Shotgun proteomics and biomarker discovery. Dis. Markers.

[4] Longuespée R., Casadonte R., Schwamborn K., Reuss D., Kazdal D., Kriegsmann K., von Deimling A., Weichert W., Schirmacher P., Kriegsmann J. (2018). Proteomics in pathology. Proteomics.

[5] Rubakhin S.S., Sweedler J.V. (2007). Characterizing peptides in individual mammalian cells using mass spectrometry. Nat. Protoc..

[6] Schwamborn K., Krieg R.C., Uhlig S., Ikenberg H., Wellmann A. (2011). Maldi imaging as a specific diagnostic tool for routine cervical cytology specimens. Int. J. Mol. Med..

[7] Amann J.M., Chaurand P., Gonzalez A., Mobley J.A., Massion P.P., Carbone D.P., Caprioli R.M. (2006). Selective profiling of proteins in lung cancer cells from fine-needle aspirates by matrix-assisted laser desorption ionization time-of-flight mass spectrometry. Clin. Cancer Res..

[8] Wang H., Kachman M.T., Schwartz D.R., Cho K.R., Lubman D.M. (2004). Comprehensive proteome analysis of ovarian cancers using liquid phase separation, mass mapping and tandem mass spectrometry: A strategy for identification of candidate cancer biomarkers. Proteomics.

[9] Hamler R.L., Zhu K., Buchanan N.S., Kreunin P., Kachman M.T., Miller F.R., Lubman D.M. (2004). A two-dimensional liquid-phase separation method coupled with mass spectrometry for proteomic studies of breast cancer and biomarker identification. Proteomics.

[10] Li H., Tang Z., Zhu H., Ge H., Cui S., Jiang W. (2016). Proteomic study of benign and malignant pleural effusion. J. Cancer Res. Clin. Oncol..

[11] Gonçalves J.P.L., Bollwein C., Schwamborn K. (2022). Mass spectrometry imaging spatial tissue analysis toward personalized medicine. Life.

[12] Czétány P., Gitta S., Balló A., Sulc A., Máté G., Szántó Á., Márk L. (2022). Application of mass spectrometry imaging in uro-oncology: Discovering potential biomarkers. Life.

[13] Caprioli R.M., Farmer T.B., Gile J. (1997). Molecular imaging of biological samples: Localization of peptides and proteins using maldi-tof ms. Anal. Chem..

[14] Walch A., Rauser S., Deininger S.-O., Höfler H. (2008). Maldi imaging mass spectrometry for direct tissue analysis: A new frontier for molecular histology. Histochem. Cell Biol..

[15] Kriegsmann J., Kriegsmann M., Kriegsmann K., Longuespée R., Deininger S.O., Casadonte R. (2019). Maldi imaging for proteomic painting of heterogeneous tissue structures. Proteom. Clin. Appl..

[16] McDonnell L.A., Angel P.M., Lou S., Drake R.R. (2017). Mass spectrometry imaging in cancer research: Future perspectives. Adv. Cancer Res..

[17] Kriegsmann J., Kriegsmann M., Casadonte R. (2015). Maldi tof imaging mass spectrometry in clinical pathology: A valuable tool for cancer diagnostics (review). Int. J. Oncol..

[18] Chung H.H., Huang P., Chen C.L., Lee C., Hsu C.C. (2022). Next-generation pathology practices with mass spectrometry imaging. Mass Spectrom. Rev..

[19] Basu S.S., Agar N.Y.R. (2021). Bringing matrix-assisted laser desorption/ionization mass spectrometry imaging to the clinics. Clin. Lab. Med..

[20] Kazdal D., Longuespée R., Dietz S., Casadonte R., Schwamborn K., Volckmar A.L., Kriegsmann J., Kriegsmann K., Fresnais M., Stenzinger A. (2019). Digital pcr after maldi-mass spectrometry imaging to combine proteomic mapping and identification of activating mutations in pulmonary adenocarcinoma. Proteom. Clin. Appl..

[21] Kriegsmann K., Longuespée R., Hundemer M., Zgorzelski C., Casadonte R., Schwamborn K., Weichert W., Schirmacher P., Harms A., Kazdal D. (2019). Combined immunohistochemistry after mass spectrometry imaging for superior spatial information. Proteom. Clin. Appl..

[22] Hideshima T., Anderson K.C. (2021). Signaling pathway mediating myeloma cell growth and survival. Cancers.

[23] Todoerti K., Gallo Cantafio M.E., Oliverio M. (2021). Oleil hydroxytyrosol (htol) exerts anti-myeloma activity by antagonizing key survival pathways in malignant plasma cells. Int. J. Mol. Sci..

[24] Morelli E., Gullà A., Rocca R., Federico C., Raimondi L., Malvestiti S., Agosti V., Rossi M., Costa G., Giavaresi G. (2020). The non-coding rna landscape of plasma cell dyscrasias. Cancers.

[25] Giles H.V., Wechalekar A., Pratt G. (2022). The potential role of mass spectrometry for the identification and monitoring of patients with plasma cell disorders: Where are we now and which questions remain unanswered?. Br. J. Haematol..

[26] Hughes C.S., McConechy M.K., Cochrane D.R., Nazeran T., Karnezis A.N., Huntsman D.G., Morin G.B. (2016). Quantitative profiling of single formalin fixed tumour sections: Proteomics for translational research. Sci. Rep..

[27] Slany A., Haudek-Prinz V., Meshcheryakova A., Bileck A., Lamm W., Zielinski C., Gerner C., Drach J. (2014). Extracellular matrix remodeling by bone marrow fibroblast-like cells correlates with disease progression in multiple myeloma. J. Proteome Res..

[28] Glavey S.V., Naba A., Manier S., Clauser K., Tahri S., Park J., Reagan M.R., Moschetta M., Mishima Y., Gambella M. (2017). Proteomic characterization of human multiple myeloma bone marrow extracellular matrix. Leukemia.

[29] Zhang H.T., Tian E.B., Chen Y.L., Deng H.T., Wang Q.T. (2015). Proteomic analysis for finding serum pathogenic factors and potential biomarkers in multiple myeloma. Chin. Med. J..

[30] Späth F., Wibom C., Krop E.J.M., Santamaria A.I., Johansson A.S., Bergdahl I.A., Hultdin J., Vermeulen R., Melin B. (2019). Immune marker changes and risk of multiple myeloma: A nested case-control study using repeated pre-diagnostic blood samples. Haematologica.

[31] Łuczak M., Kubicki T., Rzetelska Z., Szczepaniak T., Przybyłowicz-Chalecka A., Ratajczak B., Czerwińska-Rybak J., Nowicki A., Joks M., Jakubowiak A. (2017). Comparative proteomic profiling of sera from patients with refractory multiple myeloma reveals potential biomarkers predicting response to bortezomib-based therapy. Pol. Arch. Intern. Med..

[32] Mills J.R., Kohlhagen M.C., Dasari S., Vanderboom P.M., Kyle R.A., Katzmann J.A., Willrich M.A., Barnidge D.R., Dispenzieri A., Murray D.L. (2016). Comprehensive assessment of m-proteins using nanobody enrichment coupled to maldi-tof mass spectrometry. Clin. Chem..

[33] Murray D.L., Puig N., Kristinsson S., Usmani S.Z., Dispenzieri A., Bianchi G., Kumar S., Chng W.J., Hajek R., Paiva B. (2021). Mass spectrometry for the evaluation of monoclonal proteins in multiple myeloma and related disorders: An international myeloma working group mass spectrometry committee report. Blood Cancer J..

[34] Amodio N., Stamato M.A., Juli G., Morelli E., Fulciniti M., Manzoni M., Taiana E., Agnelli L., Cantafio M.E.G., Romeo E. (2018). Drugging the lncrna malat1 via lna gapmer aso inhibits gene expression of proteasome subunits and triggers anti-multiple myeloma activity. Leukemia.

[35] Juli G., Oliverio M. (2019). Anti-tumor activity and epigenetic impact of the polyphenol oleacein in multiple myeloma. Cancers.

[36] Boonstra H., Oosterhuis J.W., Oosterhuis A.M., Fleuren G.J. (1983). Cervical tissue shrinkage by formaldehyde fixation, paraffin wax embedding, section cutting and mounting. Virchows Archiv. A Pathol. Anat. Histopathol..

[37] Hobro A.J., Smith N.I. (2017). An evaluation of fixation methods: Spatial and compositional cellular changes observed by raman imaging. Vib. Spectrosc..

[38] Chiesa G., Kiriakov S., Khalil A.S. (2020). Protein assembly systems in natural and synthetic biology. BMC Biol..

[39] Molodenskiy D., Shirshin E., Tikhonova T., Gruzinov A., Peters G., Spinozzi F. (2017). Thermally induced conformational changes and protein-protein interactions of bovine serum albumin in aqueous solution under different ph and ionic strengths as revealed by saxs measurements. Phys. Chem. Chem. Phys..

[40] Kubicki T., Bednarek K., Kostrzewska-Poczekaj M., Luczak M., Lewandowski K., Gil L., Jarmuz-Szymczak M., Dytfeld D. (2022). Bortezomib- and carfilzomib-resistant myeloma cells show increased activity of all three arms of the unfolded protein response. Am. J. Cancer Res..

[41] Zaal E.A., Wu W., Jansen G., Zweegman S., Cloos J., Berkers C.R. (2017). Bortezomib resistance in multiple myeloma is associated with increased serine synthesis. Cancer Metab..

