# Supplementary material for: A Comparison of Different Sample Processing Protocols for MALDI Imaging Mass Spectrometry Analysis of Formalin-Fixed Multiple Myeloma Cells
Source: Cancers (Basel). 2023 Feb 3;15(3):974. doi: 10.3390/cancers15030974 (PMC9913598; doi:10.3390/cancers15030974)
Supplement: Supplementary file 1 [file cancers-15-00974-s001.zip › cancers-2150668-supplementary.pdf]

A) B)

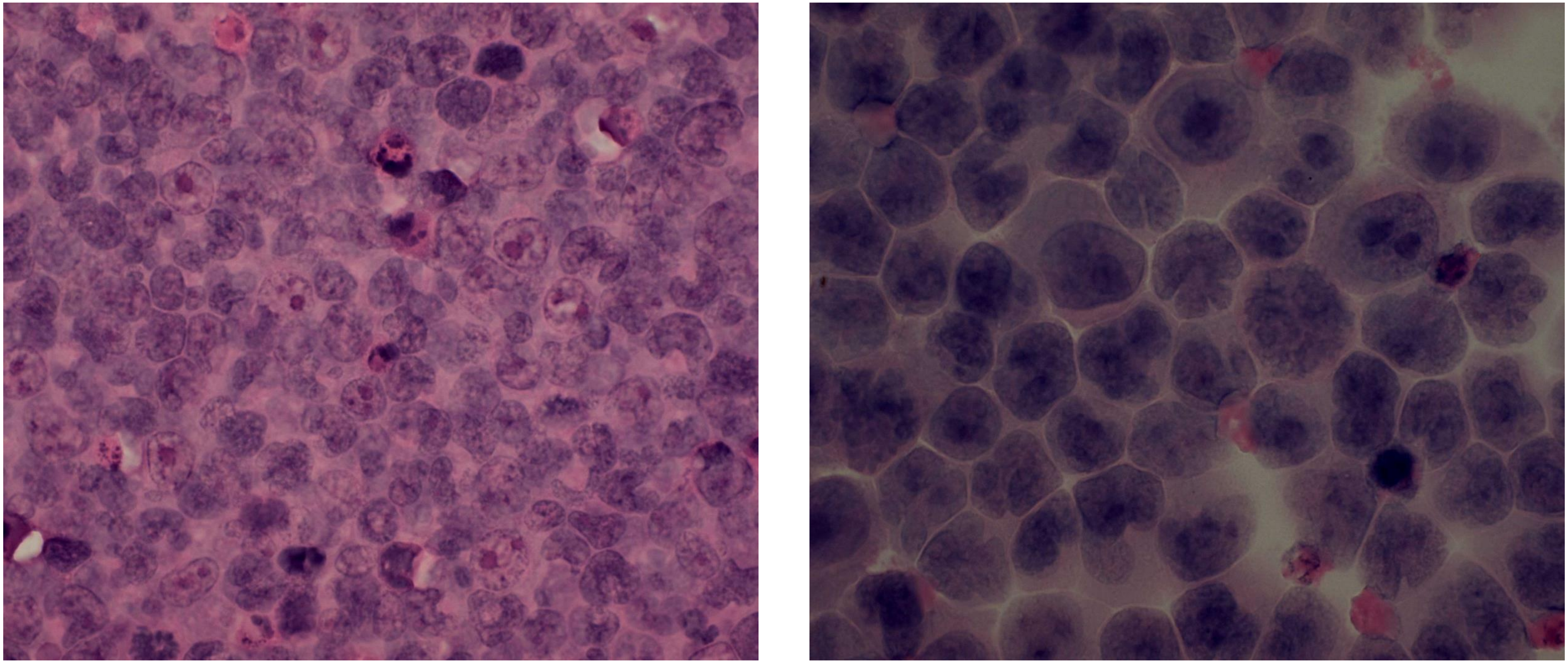

Figure S1. HE stained cells are represented for comparison between cells embedded in paraffin A) and cells centrifuged with cytospin procedure B). 100x magnification.

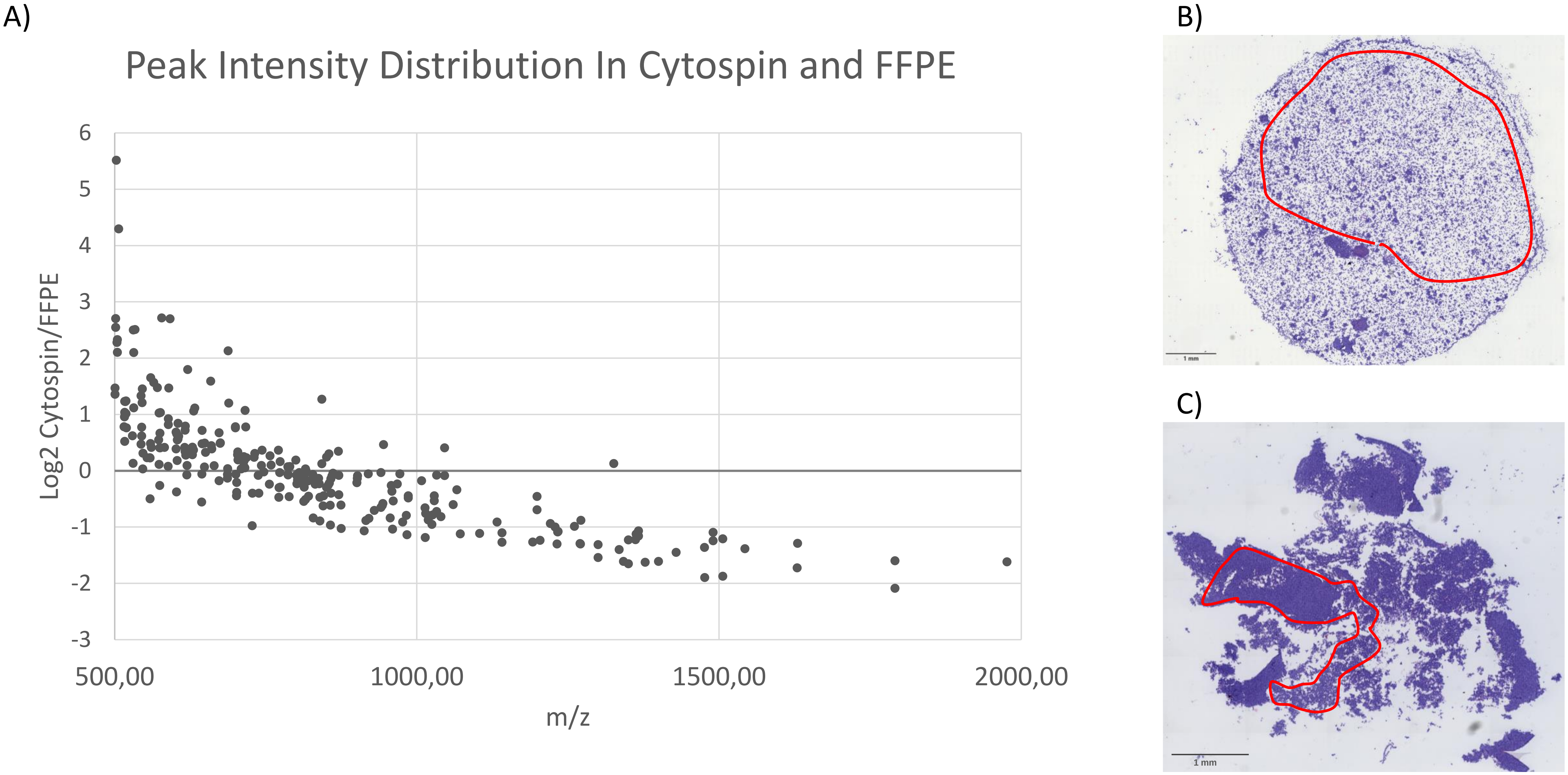


Figure S2. Peak intensity distribution in cytospin and FFPE samples through the m/z range 500-2000. A) Plot of log2 cytospin/FFPE intensity (y-axis) vs. the mass range m/z 500-2000 (x-axis) showing the peak height distribution (points in dark gray) in the m/z range considered, observed in the mean of 69 spectra from each cytospin and FFPE samples. B) Hematoxylin & Eosin staining of a cytospin sample showing a layer of sparsely packed cells. C) Hematoxylin & Eosin staining of a FFPE sample showing a layer of cells densely packed. Red lines in B) and C) indicate cell areas where the 69 spectra were randomly chosen.

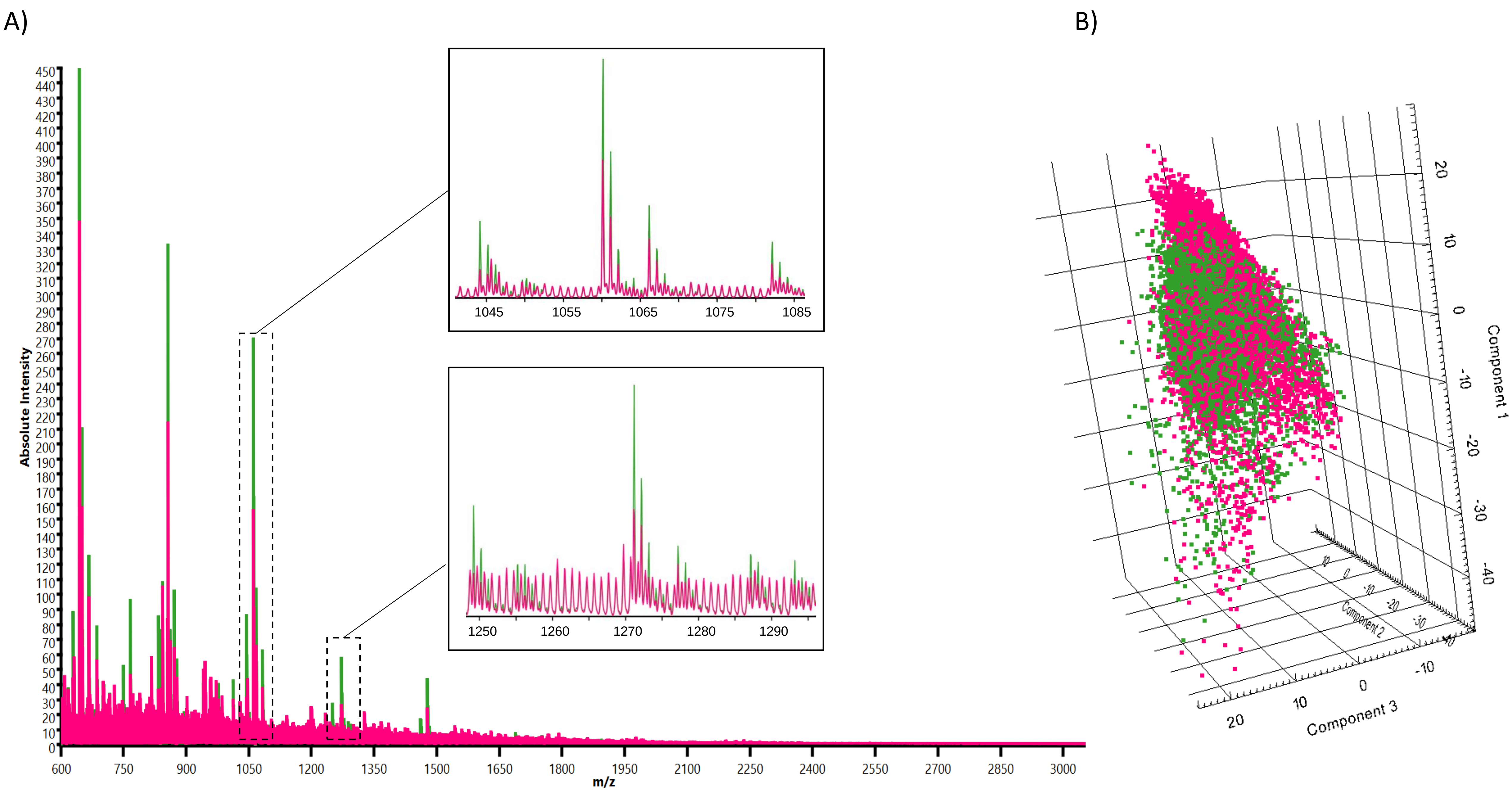


Figure S3: Peptide profiles and principal component analysis (PCA) obtained from two FFPE cell blocks prepared with two automatic processing system. A) Average mass spectrum overlay between FFPE tissue prepared with Sakura Xpress (green) and Sakura VIP (magenta) system. Mass spectra comparison shows similar peptide profile, with several mass intensities higher in the Sakura Xpress compared the Sakura VIP profile. Insets show enlarged m/z areas where many peak intensity differences between Sakura Xpress and Sakura VIP procedures are visible. B) PCA shows a scores plot between spectra acquired from FFPE tissue processed with Sakura Xpress (green) and Sakura VIP (magenta) system. No cluster segregation is observed between the two types of procedures. Each mark in the plot corresponds to a spectrum in the dataset.

**Table S1**. Sample paraffin processing protocol

| Reagent | Time (min) | Temperature (°C) | Pressure |
|---|---|---|---|
| Formalin | 2 | 40 | Atm |
| Ethanol 70% | 3 | 45 | Atm |
| Ethanol 70% | 3 | 45 | Atm |
| Isopropanol/Ethanol 80/20 | 2 | 45 | Atm |
| Isopropanol/Ethanol 80/20 | 10 | 45 | Atm |
| Isopropanol 100% | 2 | 45 | Atm |
| Isopropanol 100% | 10 | 50 | Atm |
| Isopropanol 100% | 0 | 50 | Atm |
| Paraffin | 5 | 63 | Vacuum |
| Paraffin | 5 | 63 | Vacuum |
| Paraffin | 10 | 63 | Vacuum |
| Paraffin | 15 | 63 | Vacuum |

**Table S2**. Comparison of peptide ions (m/z) intensity between Cytospin and FFPE dataset.

| m/z | Mean Intensity Cytospin | Mean Intensity FFPE | og2 Cytospin/ FFI | Total number of m/z with Log2 Intensity Cytospin /FFPE > 0 | Total number of m/z with Log2 Intensity Cytospin /FFPE < 0 |
|---|---|---|---|---|---|
| 500,5 | 6255,051163 | 2259,316667 | 1,469135191 | 124 | 145 |
| 500,6 | 5452 | 2123,933333 | 1,360047079 | | |
| 501,5 | 17138,1 | 2633,033333 | 2,702409485 | | |
| 501,6 | 14486,62609 | 2476,1 | 2,548580147 | | |
| 502,6 | 87177,5439 | 1904,5 | 5,516472365 | | |
| 503,6 | 16691,375 | 3439,4 | 2,278873991 | | |
| 504,5 | 9968,523529 | 1984,2 | 2,328822387 | | |
| 504,6 | 10160,81364 | 2364,7 | 2,10328686 | | |
| 506,6 | 55555,493 | 2825,1 | 4,297555723 | | |
| 515,4 | 12519,75897 | 7277,9875 | 0,78259531 | | |
| 516,3 | 5829,775 | 2999,2 | 0,958862473 | | |
| 516,4 | 5265,06 | 3661,875 | 0,523867427 | | |
| 516,5 | 7321,336842 | 3567,435 | 1,037219958 | | |
| 516,6 | 7802,84 | 3323,883333 | 1,231129572 | | |
| 517,6 | 4961,806667 | 2114,8 | 1,230344291 | | |
| 518,5 | 5718,031884 | 2419,86 | 1,240595081 | | |
| 518,6 | 5446,803704 | 2698,4 | 1,013305652 | | |
| 519,5 | 5637,74 | 3327,2 | 0,760808355 | | |
| 529,4 | 4178,433333 | 2713,085714 | 0,623027491 | | |
| 530,4 | 5184,7 | 4724,926667 | 0,133968575 | | |
| 530,6 | 19335,79 | 3418,772222 | 2,499723499 | | |
| 531,5 | 7068,7 | 3254,466667 | 1,119023777 | | |
| 531,6 | 9734,607778 | 2271,2 | 2,099668098 | | |
| 533,6 | 10673,79899 | 1874,75 | 2,509303619 | | |
| 543,4 | 5203,591304 | 2067,8 | 1,331411002 | | |
| 543,5 | 4109,1 | 2961,28 | 0,472601532 | | |

| | | | | | |
|---|---|---|---|---|---|
| 544,5 | 4261,007143 | 2776,35 | 0,618005018 | | |
| 544,6 | 5027,304255 | 2943,436364 | 0,772283567 | | |
| 545,5 | 4953,1 | 2141,133333 | 1,20995711 | | |
| 545,6 | 5647,671277 | 2060,2 | 1,454871721 | | |
| 546,3 | 3530,3 | 3449,75 | 0,033298972 | | |
| 546,6 | 3618,6 | 2914,425 | 0,312220365 | | |
| 553,4 | 2902,4 | 2473,425 | 0,230736207 | | |
| 558,3 | 3475,9 | 2959 | 0,232276881 | | |
| 558,4 | 3665,9 | 5175,96 | -0,497659036 | | |
| 558,6 | 4465 | 3817,836842 | 0,225904726 | | |
| 559,3 | 4799,5 | 3431,3 | 0,48412885 | | |
| 559,6 | 9243,932 | 2936,5 | 1,654409009 | | |
| 560,6 | 3864 | 2899,666667 | 0,41420803 | | |
| 564,6 | 7148,25 | 2411,3 | 1,567778938 | | |
| 570,6 | 7217,68 | 2594,133333 | 1,476282549 | | |
| 572,6 | 4539,428571 | 3102,9 | 0,548893498 | | |
| 573,4 | 3425,3 | 3163,4 | 0,114754359 | | |
| 573,6 | 5994,206 | 2946,025 | 1,024798992 | | |
| 574,4 | 3292,3 | 2485,15 | 0,405762869 | | |
| 574,6 | 2721,2 | 3262,75 | -0,261845455 | | |
| 574,7 | 3545,6 | 2230,9 | 0,66840394 | | |
| 575,3 | 5312,45 | 4000,9 | 0,409052788 | | |
| 575,6 | 5253,947917 | 2567,666667 | 1,032943974 | | |
| 577,6 | 14155,19293 | 2154,6 | 2,715839449 | | |
| 582,3 | 8243,707071 | 6175,366667 | 0,416768441 | | |
| 588,4 | 3264 | 3093,866667 | 0,077230034 | | |
| 588,6 | 4985,7 | 2629,966667 | 0,922751561 | | |
| 588,7 | 4231,45 | 2397,9 | 0,819380624 | | |
| 589,6 | 4962,039506 | 1792 | 1,469362584 | | |
| 591,6 | 17187,64235 | 2647,575 | 2,698628204 | | |
| 601,6 | 4749,706667 | 2951,7 | 0,686292319 | | |

| | | | | | |
|---|---|---|---|---|---|
| 601,7 | 3878,266667 | 2963,683333 | 0,3880207 | | |
| 602,4 | 3586,2 | 4650,8 | -0,375022952 | | |
| 602,6 | 4415,25 | 2799,15 | 0,657506329 | | |
| 603,4 | 4005,5 | 3535,328571 | 0,180138039 | | |
| 603,6 | 4511,4 | 3080,246154 | 0,550529561 | | |
| 603,7 | 3768,1 | 2574,114286 | 0,549761145 | | |
| 604,6 | 4695,646809 | 2615,7 | 0,844126813 | | |
| 604,7 | 4191,5 | 2836,85 | 0,56317676 | | |
| 605,7 | 4076,857143 | 2665,9 | 0,612834739 | | |
| 615,6 | 3835,066667 | 2873,857143 | 0,416263309 | | |
| 616,4 | 3922,233333 | 3091,766667 | 0,34324392 | | |
| 616,6 | 4830,66 | 2934,655556 | 0,719029132 | | |
| 616,7 | 5434,302105 | 3134,857143 | 0,79369507 | | |
| 617,7 | 3299,5 | 2721,18 | 0,278015027 | | |
| 619,4 | 4409,8375 | 4638,8 | -0,073026152 | | |
| 619,7 | 4692 | 4394,371429 | 0,094546197 | | |
| 620,6 | 6931,4 | 1990,7 | 1,799870954 | | |
| 628,6 | 4123,5 | 3388,13125 | 0,283379644 | | |
| 628,7 | 4659,675 | 3488,614286 | 0,417575238 | | |
| 630,4 | 5919,173684 | 4578,333333 | 0,370573285 | | |
| 630,7 | 8242,131 | 3954,058824 | 1,05968306 | | |
| 632,6 | 3953,4 | 1826,3 | 1,114170161 | | |
| 644 | 6010,452941 | 8840,5 | -0,556654253 | | |
| 644,1 | 5790,6 | 6033,509091 | -0,059284476 | | |
| 644,4 | 4466,9 | 3202 | 0,48030065 | | |
| 644,7 | 4509,014103 | 2745,9 | 0,715532936 | | |
| 648,4 | 3522,8 | 3359,766667 | 0,068361529 | | |
| 648,7 | 4242,823256 | 3021,316667 | 0,489847179 | | |
| 650,1 | 6204,330303 | 4948,816667 | 0,326191897 | | |
| 658,7 | 6829,604211 | 2265,35 | 1,592068008 | | |
| 659,7 | 3952,090909 | 2901,5 | 0,445817204 | | |

| | | | | | |
|---|---|---|---|---|---|
| 660,7 | 3977,785714 | 3041 | 0,387419743 | | |
| 664,4 | 5068,374713 | 4764,38 | 0,089234704 | | |
| 672,5 | 3084,05 | 3488,7 | -0,177863388 | | |
| 672,7 | 4054,425 | 2542,333333 | 0,673344126 | | |
| 674,4 | 4127,833333 | 2941,1 | 0,489028883 | | |
| 674,7 | 3798,26 | 2697,5 | 0,493715705 | | |
| 686,4 | 4072,966667 | 4461,7 | -0,131513504 | | |
| 686,7 | 3752,6 | 3924,930769 | -0,064776689 | | |
| 686,8 | 3637,233333 | 3545 | 0,037055852 | | |
| 687,7 | 11245,20455 | 2569,575 | 2,129708237 | | |
| 688,7 | 4651,85 | 2022,7 | 1,201522217 | | |
| 699,7 | 5418,225 | 3193,416667 | 0,762719502 | | |
| 699,8 | 5432,619277 | 3157,53 | 0,782851504 | | |
| 700,5 | 3292,78 | 3439,35 | -0,062829812 | | |
| 701,5 | 3944,85 | 5365,935714 | -0,443859327 | | |
| 701,7 | 4504,268966 | 5198,795652 | -0,20688447 | | |
| 701,8 | 4378,946296 | 5716,43 | -0,384530686 | | |
| 703,4 | 4558,202564 | 3744,6 | 0,283653421 | | |
| 703,7 | 4483,522222 | 3551,706667 | 0,336120113 | | |
| 703,8 | 4365,212281 | 3814,2 | 0,19467132 | | |
| 709,4 | 4507,017647 | 4405,85 | 0,03275272 | | |
| 713,4 | 3553,878261 | 3359,7 | 0,081061847 | | |
| 713,7 | 3900,2 | 3280,51 | 0,249627988 | | |
| 713,8 | 3603,475 | 3192,75 | 0,174589245 | | |
| 715,5 | 5326,863158 | 5133,876923 | 0,05323751 | | |
| 715,8 | 8812,683 | 4192,154545 | 1,071889408 | | |
| 716,7 | 3881,5 | 2266,225 | 0,776323183 | | |
| 717,8 | 3998,482609 | 3406,35 | 0,231225934 | | |
| 727,5 | 3305,9 | 6509,923077 | -0,977597411 | | |
| 727,8 | 4573,047253 | 6034,7 | -0,400126226 | | |
| 730,8 | 3764 | 3188,8 | 0,239253013 | | |

| | | | | | |
|---|---|---|---|---|---|
| 731,7 | 3617,1 | 2918,833333 | 0,309441649 | | |
| 737,4 | 4160,233333 | 4408,1 | -0,083492506 | | |
| 738,4 | 3590,4 | 4737,35 | -0,399935683 | | |
| 742,8 | 3797,35 | 3561,388889 | 0,092552997 | | |
| 743,8 | 4602,544048 | 3577 | 0,36368141 | | |
| 746,5 | 3917,5 | 3978,5 | -0,022291324 | | |
| 755,8 | 3428,04 | 4045,1 | -0,238791425 | | |
| 756,8 | 3924,71875 | 3266,742857 | 0,264736375 | | |
| 758,8 | 3862,438889 | 3595,976923 | 0,103128345 | | |
| 770,8 | 3725,99 | 2893,3 | 0,364907881 | | |
| 771,5 | 3517,028571 | 4870,222222 | -0,469630547 | | |
| 771,8 | 3187,575 | 3924,317647 | -0,299982535 | | |
| 772,8 | 3956,203226 | 4045,875 | -0,03233521 | | |
| 773,5 | 3003,371429 | 3675,314286 | -0,291284719 | | |
| 773,8 | 3904,55 | 3492,345455 | 0,160960013 | | |
| 786,5 | 3379,831579 | 3219,966667 | 0,069905604 | | |
| 786,8 | 3845,425 | 4043,673333 | -0,072523399 | | |
| 786,9 | 3448,8 | 3602,15 | -0,062763791 | | |
| 788,5 | 3903,590909 | 5369,4 | -0,459959016 | | |
| 789,5 | 3412,955556 | 3236,4 | 0,0766317 | | |
| 799,8 | 3639,2 | 3187 | 0,191422321 | | |
| 800,8 | 3511,25 | 3709,7 | -0,079317803 | | |
| 801,5 | 2850 | 3340,4 | -0,229058951 | | |
| 802,5 | 3844,142857 | 4307,05 | -0,164038121 | | |
| 802,9 | 3877,8 | 4039,8 | -0,059045473 | | |
| 805,5 | 3704,1 | 3785,333333 | -0,031297301 | | |
| 806,5 | 3137,833333 | 3641,066667 | -0,214592431 | | |
| 812,5 | 4652,976768 | 5090,366667 | -0,129615596 | | |
| 812,9 | 3115,1 | 4547,025 | -0,545644459 | | |
| 813,5 | 3177 | 3894,1 | -0,293624843 | | |
| 814,9 | 3234,033333 | 3341,45 | -0,047139738 | | |

| | | | | | |
|---|---|---|---|---|---|
| 815,5 | 3823,385185 | 3733,45 | 0,034341141 | | |
| 815,8 | 2557,8 | 3641,1 | -0,509470903 | | |
| 815,9 | 3407,7 | 3892,875 | -0,192037688 | | |
| 816,5 | 9617,904 | 9570,08 | 0,007191541 | | |
| 819,5 | 3329,7 | 3777,1 | -0,181886781 | | |
| 820,5 | 3955,5 | 5412,6 | -0,452461703 | | |
| 827,9 | 3393,9 | 3555,75 | -0,067209837 | | |
| 828,5 | 2680,5 | 4791,825 | -0,838073086 | | |
| 831,5 | 3902,130303 | 4599,25 | -0,237136666 | | |
| 831,9 | 3836,3 | 4385,7 | -0,193091585 | | |
| 837,5 | 4249,647191 | 4665,5 | -0,13468863 | | |
| 838,5 | 3555,7 | 4177,3 | -0,23243715 | | |
| 839,5 | 3733,1 | 5176,2 | -0,471519207 | | |
| 839,6 | 2225,5 | 4132,771429 | -0,892980074 | | |
| 842,5 | 16122,01667 | 6683,25 | 1,270410471 | | |
| 842,6 | 15229,51875 | 13973,91111 | 0,124134485 | | |
| 844,9 | 2468,2 | 3809,6 | -0,626180224 | | |
| 845,5 | 3514,9 | 4778,6 | -0,443104365 | | |
| 850,5 | 3773,98 | 3191,5 | 0,24185213 | | |
| 850,6 | 3170,2 | 3893,066667 | -0,296333191 | | |
| 853,5 | 3841,8 | 4586 | -0,255453939 | | |
| 854,5 | 5751 | 4668 | 0,301008277 | | |
| 855,1 | 6124,93 | 7128,26 | -0,218856603 | | |
| 856,6 | 3614,05 | 5523,4 | -0,611940144 | | |
| 856,9 | 2443,9 | 4764,8 | -0,963230405 | | |
| 857,6 | 3121,042857 | 3442 | -0,141218929 | | |
| 857,9 | 3339,4 | 4405,688889 | -0,399778709 | | |
| 859,5 | 3808,7 | 4065,7 | -0,09420511 | | |
| 861,1 | 4249,7 | 4377,9 | -0,042878 | | |
| 869,9 | 3447,075 | 2712,5 | 0,345749551 | | |
| 870,6 | 5156,895918 | 5451,583333 | -0,080172373 | | |

| | | | | | |
|---|---|---|---|---|---|
| 870,9 | 3512,9 | 4716,253333 | -0,424978706 | | |
| 874,5 | 3234,24 | 4931,8 | -0,608687556 | | |
| 874,6 | 2093 | 4260,333333 | -1,025394001 | | |
| 900,6 | 4129,636842 | 4778,62 | -0,21057913 | | |
| 901,5 | 6011,996429 | 6609,1 | -0,136609673 | | |
| 901,6 | 6084,713636 | 6468,9 | -0,088331044 | | |
| 912,6 | 2029,9 | 4253,3 | -1,06717396 | | |
| 915,6 | 2824,56 | 5217,45 | -0,885318723 | | |
| 919,5 | 3133,3 | 3256,6 | -0,05568362 | | |
| 920,5 | 2727,633333 | 4904,7 | -0,846515175 | | |
| 929,6 | 2992,9 | 4875,775 | -0,704087476 | | |
| 940,5 | 5432,563514 | 5561,25 | -0,033776057 | | |
| 940,6 | 5123,461538 | 8044,1 | -0,650812156 | | |
| 942,6 | 3011,9 | 4619,15 | -0,616953525 | | |
| 943,6 | 3886,812963 | 5820,175 | -0,582474847 | | |
| 944,6 | 14449,563 | 10483 | 0,462974219 | | |
| 955,6 | 2842,513636 | 5086,3 | -0,839449287 | | |
| 957,6 | 5994,13 | 7182,875 | -0,261011036 | | |
| 958,6 | 3281,3 | 4229,033333 | -0,36606043 | | |
| 959,6 | 2720,277778 | 5585,9 | -1,038035768 | | |
| 960,6 | 3318 | 4812,32 | -0,536418693 | | |
| 967,6 | 4781,72766 | 5622,133333 | -0,233585704 | | |
| 971,6 | 5666,575 | 5883,15 | -0,054111818 | | |
| 976,5 | 3464,227778 | 6516,075 | -0,911469417 | | |
| 982,6 | 2795,69 | 4843,45 | -0,792830652 | | |
| 983,6 | 2204,6 | 4841,8 | -1,135026569 | | |
| 985,6 | 4449,481522 | 6056,3 | -0,444799436 | | |
| 985,7 | 5205,5 | 7275,136364 | -0,482937546 | | |
| 1007,6 | 3016,85 | 3414,9 | -0,178800366 | | |
| 1013,5 | 3051,673913 | 4823,8 | -0,660569282 | | |
| 1013,6 | 1897 | 4317,85 | -1,186593447 | | |

| | | | | | |
|---|---|---|---|---|---|
| 1014,5 | 2286,95 | 3872,6 | -0,759877669 | | |
| 1018,6 | 3264,4 | 5981,3 | -0,873641232 | | |
| 1024,7 | 2515,35 | 4866,04 | -0,951989021 | | |
| 1025,7 | 2407,45 | 4145,3 | -0,78397068 | | |
| 1028,6 | 3396,587805 | 4913,3 | -0,532606181 | | |
| 1028,7 | 3838,346939 | 5217,4375 | -0,442856294 | | |
| 1032,6 | 4692,956322 | 4947,95 | -0,076333889 | | |
| 1032,7 | 3959,518182 | 6531,58 | -0,722107139 | | |
| 1039,6 | 2982,085 | 5246,6 | -0,815061423 | | |
| 1045,6 | 6955,560606 | 5238 | 0,409150738 | | |
| 1045,7 | 6285,5 | 6676,316667 | -0,087024872 | | |
| 1060,1 | 3278,035294 | 4979,7 | -0,603227443 | | |
| 1066,1 | 3696,090476 | 4669,833333 | -0,337370988 | | |
| 1071,6 | 2315,757143 | 5035,133333 | -1,120546019 | | |
| 1103,7 | 2389,233333 | 5167,55 | -1,112932688 | | |
| 1132,6 | 2567,268571 | 4824,066667 | -0,910015615 | | |
| 1140,6 | 2255,3 | 4840 | -1,101687694 | | |
| 1140,7 | 2101,9 | 5068,566667 | -1,269883794 | | |
| 1191,7 | 2255,5 | 5427,8 | -1,266920276 | | |
| 1198,7 | 4702,45 | 7597,7 | -0,692150143 | | |
| 1198,8 | 6169,01625 | 8455,8 | -0,454900807 | | |
| 1203,7 | 2308,554839 | 5440,8 | -1,236828794 | | |
| 1220,7 | 2022,02 | 3871,1 | -0,936946309 | | |
| 1228,7 | 1945,9 | 3886,4 | -0,997996823 | | |
| 1231,7 | 2368,420833 | 5832,35 | -1,300151849 | | |
| 1232,7 | 2352,802222 | 4996,9 | -1,086653295 | | |
| 1233,7 | 2131,075 | 4497,9 | -1,077670219 | | |
| 1260,7 | 2329,4 | 4620,8 | -0,98818425 | | |
| 1269,8 | 2309,907143 | 5658,2 | -1,292508315 | | |
| 1270,7 | 1905,25 | 4691 | -1,299915185 | | |
| 1271,1 | 2153,55 | 3968,7 | -0,881949691 | | |

| | | | | | |
|---|---|---|---|---|---|
| 1299,7 | 1616,6 | 4700 | -1,539698004 | | |
| 1299,8 | 1919,2 | 4769,7 | -1,313393465 | | |
| 1325,8 | 6568,983 | 6009,325 | 0,128467083 | | |
| 1334,7 | 2009,990909 | 5304,45 | -1,400014195 | | |
| 1341,8 | 1639,8 | 5005,4 | -1,609965499 | | |
| 1349,7 | 1802,033333 | 4219,8 | -1,227548925 | | |
| 1349,8 | 1882,6 | 5922 | -1,65335799 | | |
| 1361,7 | 1984,23 | 4636,7 | -1,224519122 | | |
| 1362,8 | 1958,6 | 4270,7 | -1,124649768 | | |
| 1366,6 | 2335,2 | 4896,4 | -1,068175305 | | |
| 1366,7 | 2313,992308 | 5163,3 | -1,157909356 | | |
| 1377,7 | 2080,421875 | 6413,4 | -1,624213277 | | |
| 1399,8 | 1852,866667 | 5657,1 | -1,610303606 | | |
| 1428,8 | 1909,821429 | 5217,6 | -1,449948596 | | |
| 1475,9 | 1915,30625 | 4924,5 | -1,362402159 | | |
| 1476 | 1338,8 | 4980 | -1,895205286 | | |
| 1489,9 | 2041,357895 | 4359,7 | -1,094699723 | | |
| 1490 | 2091,104167 | 4952,3 | -1,243833782 | | |
| 1505,9 | 2273,626744 | 5251,6 | -1,207761604 | | |
| 1506 | 1041,5 | 3814,1 | -1,87267983 | | |
| 1542,8 | 1994,196552 | 5201,666667 | -1,38316634 | | |
| 1628,9 | 1523,809091 | 5036,6 | -1,724767991 | | |
| 1629,9 | 1357,2 | 3317,8 | -1,289593587 | | |
| 1790,9 | 1582,181818 | 4789,9 | -1,598080139 | | |
| 1791 | 1548,028571 | 6583,45 | -2,088411715 | | |
| 1976,1 | 1336,090909 | 4105 | -1,619364048 | | |

* Number of spectra used for each dataset = 69.
